# Brain-cognition relationships and treatment outcome in treatment-resistant late-life depression

**DOI:** 10.21203/rs.3.rs-6340032/v1

**Published:** 2025-06-03

**Authors:** Aristotle Voineskos, Peter Zhukovsky, Meryl Butters, Helen Lavretsky, Patrick Brown, Joshua Shimony, Eric Lenze, Daniel Blumberger, Alastair Flint, Jordan Karp, Steven Roose, Erin Dickie, Daniel Felsky, Ginger Nichol, Yar Al-Dabagh, Nicole Schoer, Feyi Obiri, Ashlyn Runk, Kayla Conaty, Benoit H. Mulsant

**Affiliations:** CAMH; Harvard Medical School/McLean Hospital; University of Pittsburgh School of Medicine; UCLA; Columbia; Washington University; Washington University School of Medicine; Centre for Addiction and Mental Health; UHN; University of Arizona; Columbia; CAMH; Centre for Addiction and Mental Health; Washington University; CAMH; CAMH; CAMH; LSU; UPMC; Department of Psychiatry, University of Toronto

## Abstract

Older adults with treatment-resistant depression are at significant risk for cognitive impairment. The relationship between treatment response and cognitive function in this population is not well-established. We examined neural correlates of executive and memory function, and their relationship with prospective treatment outcomes. In the context of a longitudinal biomarker study embedded within a multi-center randomized controlled trial for late-life treatment-resistant depression, 397 participants completed baseline neuropsychological testing, and of these 234 adults successfully completed a baseline MRI scan. Multivariate regressions were used to test for brain-cognition associations between memory and executive function and brain functional connectivity, white matter integrity, and gray matter structure. Further, we employed regularized elastic net regressions to identify biomarkers predicting depression remission (MADRS≤10) in the clinical trial. Among participants who completed neuroimaging better cognition was associated with lower connectivity between components of the default mode and the frontoparietal networks and within the frontoparietal network (multivariate r=0.37, p<0.01). Using diffusion imaging data, lower tract integrity in a distributed set of tracts was associated with poorer executive function (multivariate r=0.27, p<0.05). Additionally, gray matter structure was positively associated with cognition (multivariate r=0.38, p<0.05). Education and better structural brain maintenance but not overall health were associated with better cognition. Ongoing treatment resistance was predicted by poorer cognition and gray matter structure. We identified distinct cross-sectional associations between specific neural circuits and variation in cognitive function in people with treatment-resistant late-life depression. We also found worse cognitive function and gray matter structure predicted ongoing treatment resistance to medication offered in the clinical trial.

## INTRODUCTION

Treatment-resistant depression (LLD) is a chronic, debilitating disorder defined by the presence of persistent depressive symptoms despite two adequate trials of antidepressant medications. It presents a multitude of challenges in treatment, as less than one-third of patients with treatment-resistant LLD remit, even in augmentation treatment trials^1–3^. In addition, high rates of cognitive impairment in this population significantly increase the risk of dementia^4^, with up to 4–6 fold increases following a recent LLD episode (i.e., within 10 years)^5^. Conversely, remission may be protective from cognitive impairment as remitted LLD patients do not show significant differences in brain structure and function from healthy controls^6^. Impairment in executive function and episodic memory are found in both LLD and in early dementia. Similarly, frontal executive and cortico-limbic circuits underpinning those key cognitive functions are also impaired in both LLD and dementia^7^.

The shared circuitry underlying LLD and dementia includes hippocampal and cortico-limbic changes^8^ as well as fronto-executive dysfunction, potentially via frontostriatal ischemia^7^. Biomarkers common to both LLD and dementia have been identified using structural^9^, functional, and diffusion magnetic resonance imaging (MRI). Although findings in case-control studies of LLD are highly heterogeneous^8,10,11^, network mapping based approaches have localized structural differences in major depression to frontoparietal, dorsal attention and visual networks, some of which also encompass posterior parietal and medial temporal areas affected in early Alzheimer’s Disease^12,13^. Further, brain-cognition studies identify lower connectivity between frontoparietal and default mode networks and worse executive function and memory in older adults with depressive symptoms^14^ and non-depressed older adults with varying levels of cognitive function^15^. Finally, disruption of axonal white matter tracts, measured using white matter hyperintensities^16,17,18^ has been shown in both dementia and LLD^19,20,21^. However, there is a paucity of studies investigating neural mechanisms linking cognitive impairment and depression neurobiology in LLD, and no well-powered clinical trials to date have prospectively collected a wide breadth of precision biomarkers in treatment-resistant LLD.

Here, we present an analysis of the baseline neuroimaging and cognitive data from the Optimizing Outcomes of Treatment-Resistant Depression in Older Adults – Neurocognitive and Neuroimaging Biomarkers (OPTIMUM-NEURO) study, where we investigate the neural correlates and protective factors for cognitive function in these high-risk older individuals. These participants also participated in the ‘OPTIMUM’ clinical trial^1^, which evaluated antidepressant switch and augmentation strategies. In functional connectivity analyses, we focus on large scale networks derived from the UK Biobank^22^, and leverage state-of-the art white matter tractography to assess brain-wide tract integrity in relation to cognitive function. We hypothesized that loss of gray and white matter alongside with de-segregation of the executive-control and cortico-limbic circuits will be associated with worse cognitive performance in cross-sectional analyses. We also expected education, a known proxy for cognitive reserve and resilience^23^, to show protective effects on brain circuits and cognition. Finally, we tested the ability of baseline imaging and cognitive data to predict acute treatment outcomes in the OPTIMUM clinical trial, expecting the addition of neuroimaging features to improve prediction performance.

## RESULTS

The sample was predominantly female, and we observed a wide range of neuropsychological performance, with over 40% of the sample assigned a neuropsychological diagnosis of MCI (Table 1).

### FC patterns associated with cognitive function

**Figure 2:**
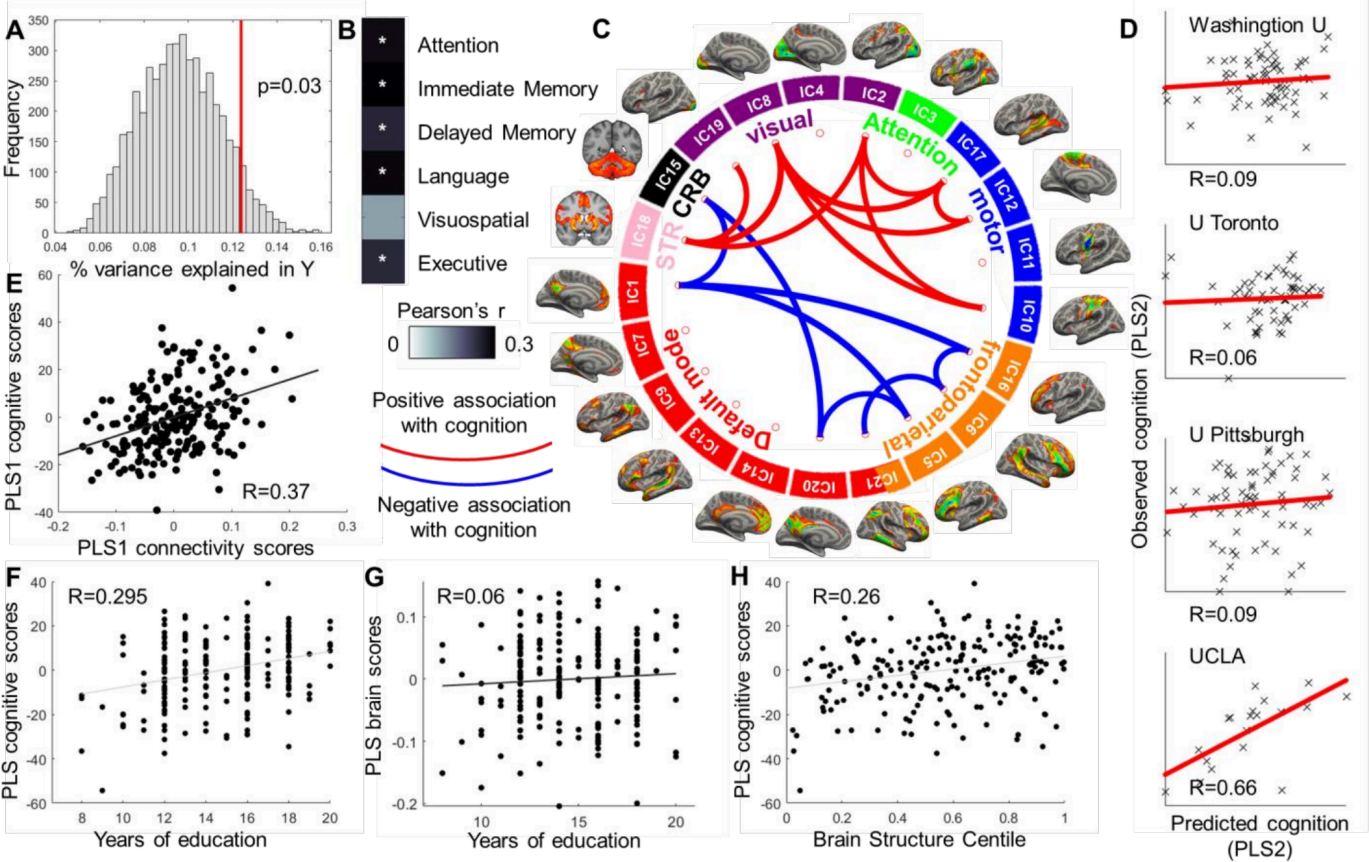
Associations between resting-state functional connectivity (FC) and cognitive function. Lower FC between DMN and FPN components was significantly associated with better cognitive performance (A, permutation distribution is shown in gray and the observed value in red; p=0.03). Higher cognitive scores on FC-PLS1 were associated with better cognitive performance on a range of cognitive tests (E, B, P_BONFERRONI_<0.05). Lower connectivity of frontoparietal and default mode network components with each other (shown in blue), and higher connectivity of visual network components (shown in red) significantly contributed to FC-PLS1 brain scores (|Z|>3) and was associated with better cognitive performance (C). Further, we observed good generalizability of the results in hold-out samples (D). Data were trained on three of the four sites. We indicate held-out sites used for model testing in (D). Education (F) and brain structure centile scores (H) had protective effects on cognitive function measured as the PLS latent variable cognitive scores. FC-PLS: functional connectivity partial least squares regression.

A PLS regression model with two latent variables showed that functional connectivity explained 8.4% of variance in 12 cognitive tests, significantly more than expected by chance (p_PERMUTATION_<0.05, Fig. 2A). The model included two latent variables. The first latent variable (FC-PLS1) was significantly correlated with attention, immediate and delayed memory, language and executive function (Fig. 2B). We found that greater connectivity of the DMN with the FPN (e.g., between independent components (IC) 1 and 5) were robustly associated with worse cognitive performance across multiple domains. More details on IC definition can be found in the Supplementary Information. Similarly, lower connectivity of different FPN components with each other was also robustly associated with cognitive performance. Higher connectivity of visuo-motor connectivity was associated with better cognitive performance. In total, 17 connectivities showed significant loadings on FC-PLS1 (Fig. 2C). Supplementary FC analyses with a precuneus seed provided a more specific brain connectivity map linked to cognitive function. A generalizability analysis of held-out data from each of the four sites showed modest generalizability in all sites except for UCLA that showed higher generalizability (Fig. 2D). Finally, we found that better cognitive performance summarized by the FC-PLS1 cognitive scores was significantly associated with years of education (p = 1.3×10^−5^) and with brain structure centile (p = 0.00016), but not with ATHF (r= −0.08, p = 0.26). The second latent variable (FC-PLS2) captured a large amount of variance in the FC data, but was not significantly associated with cognition.

### Reduced fractional anisotropy associated with worse executive function

**Figure 3:**
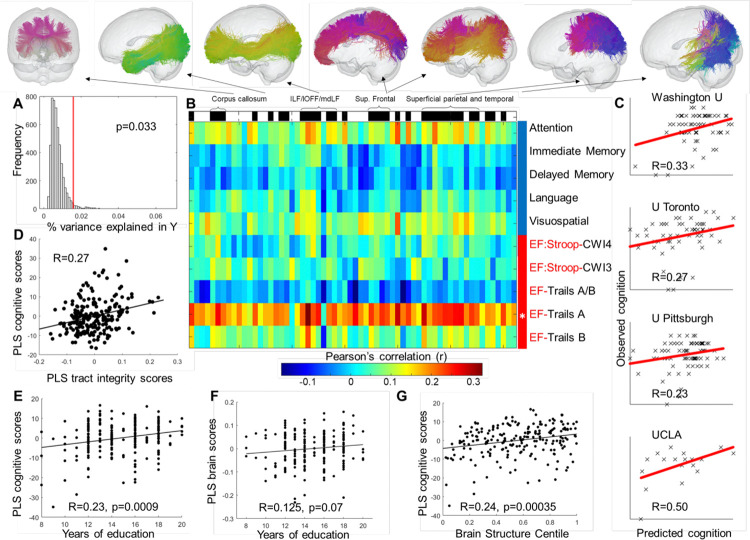
Associations between tract integrity measured using fractional anisotropy (FA) and cognitive function. Higher FA in a range of tracts including corpus callosum, longitudinal tracts, superior frontal and superficial tracts was associated with better executive function, as the overall PLS model explained a significantly larger amount of variance than expected by chance (A, permutation distribution is shown in gray and the observed value in red; p=0.033). Univariate Pearson’s correlations between individual cognitive tests and tracts are shown in (B), with tracts that significantly contributed to WM-PLS1 (|Z|>3) highlighted in black on the top of the panel. One motor and executive function test (Trails A) significantly contributed to WM-PLS1 (*P_BONFERRONI_<0.05). Multivariate correlation between cognitive WM-PLS1 scores and white matter integrity WM-PLS1 scores is shown in (D). We observed good generalizability of the results for Trails A performance in all hold-out samples (C). Education (E) and brain structure centile scores (F) had protective effects on executive function measured as the PLS latent variable cognitive scores. We did not observe significant associations between clinical measures of treatment resistance at baseline with PLS latent variable scores (G). WM-PLS: white matter partial least squares regression.

A PLS regression model testing for a relationship between FA in 62 tracts and cognition explained 1.6% of variance in all cognitive tests, significantly more than expected by chance (p_PERMUTATION_<0.05, Fig. 3A) as FA data specifically predicted Trails A performance but not the performance on any other cognitive test. The model included one latent variable (WM-PLS1), which was significantly correlated with the Trails-A executive function test (Fig. 3B). In total, 33 tracts significantly contributed to the WM-PLS1, with lower FA in those tracts predicting lower executive function. A prediction analysis in held-out data showed moderate-to-high generalizability in all sites (Fig. 3E). Finally, we found that better cognitive performance summarized by the WM-PLS1 cognitive scores was significantly associated with years of education (p = 0.0009) and with brain structure centile (Fig. 3F, p = 0.00035), but not with resistance to antidepressant treatment (Fig. 3G, ATHF r=−0.08, p = 0. 23). White matter hyperintensities were significantly associated with both WM-PLS1 scores representing white matter integrity (r = 0.397, p = 1.6×10^−9^) and with the cognitive scores (r = 0.17, p = 0.01).

### Regional brain structure associated with cognitive function

**Figure 4:**
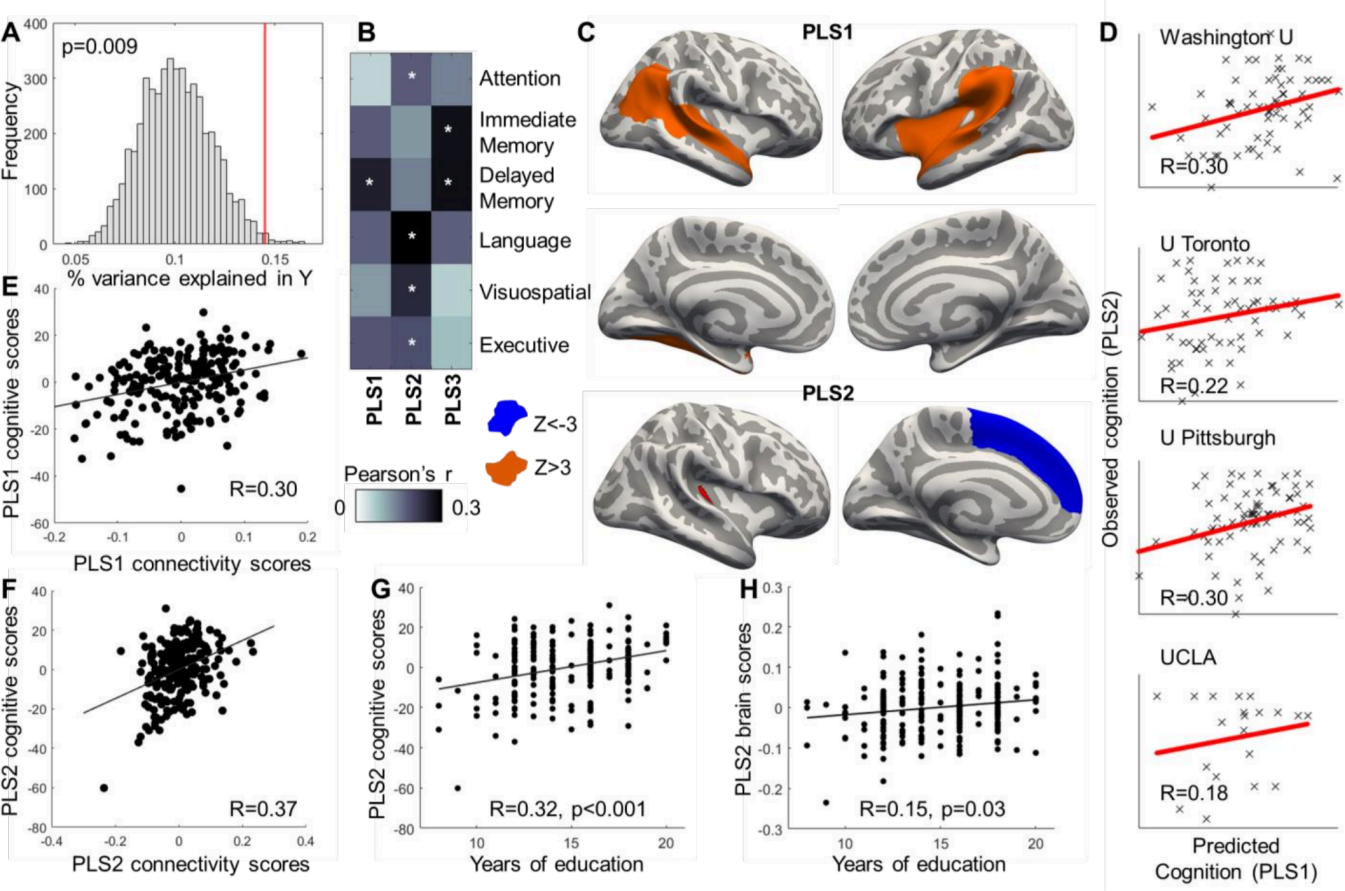
Associations between brain structure and cognitive function. Higher cortical thickness in the insular cortex and greater volume of the hippocampus were associated with better cognitive performance in a range of cognitive tests, as the overall PLS model explained a significantly larger amount of variance than expected by chance (A, permutation distribution is shown in gray and the observed value in red; p=0.009). Univariate Pearson’s correlations between individual cognitive tests and latent variable scores are shown in (B, *P_BONFERRONI_<0.05), with regions that significantly contributed to GM-PLS1 and GM-PLS2 (|Z|>3) shown in (C). Higher cortical thickness of the insular cortex and medial temporal regions (shown in orange) and normalized volumes of the bilateral hippocampus were positively associated with cognitive function, while thickness of the superior frontal gyrus was negatively associated with cognition (shown in blue). Multivariate correlation between brain structure scores and cognitive scores for GM-PLS1 and GM-PLS2 are shown in (E) and (F), respectively. We observed good generalizability of the results in most hold-out samples (D). Education had protective effects on cognitive function (G) and brain structure latent scores (H). GM-PLS: gray matter partial least squares regression.

A PLS regression model testing for a relationship between cortical and subcortical brain structure and cognition explained 14.5% of variance in cognitive function, significantly more than expected by chance (p_PERMUTATION_<0.05, Fig. 4A). The PLS model generated three latent variables. Two of those variables (GM-PLS1 and GM-PLS3) were significantly correlated with memory performance (Fig. 4B, Bonferroni P < 0.05). GM-PLS2 was significantly correlated with the non-memory related cognitive domains. We did not observe significant associations between clinical measures of resistance to antidepressant treatment at baseline (ATHF) with PLS latent variable scores.

### Identifying Markers predicting treatment outcomes

**Figure 5:**
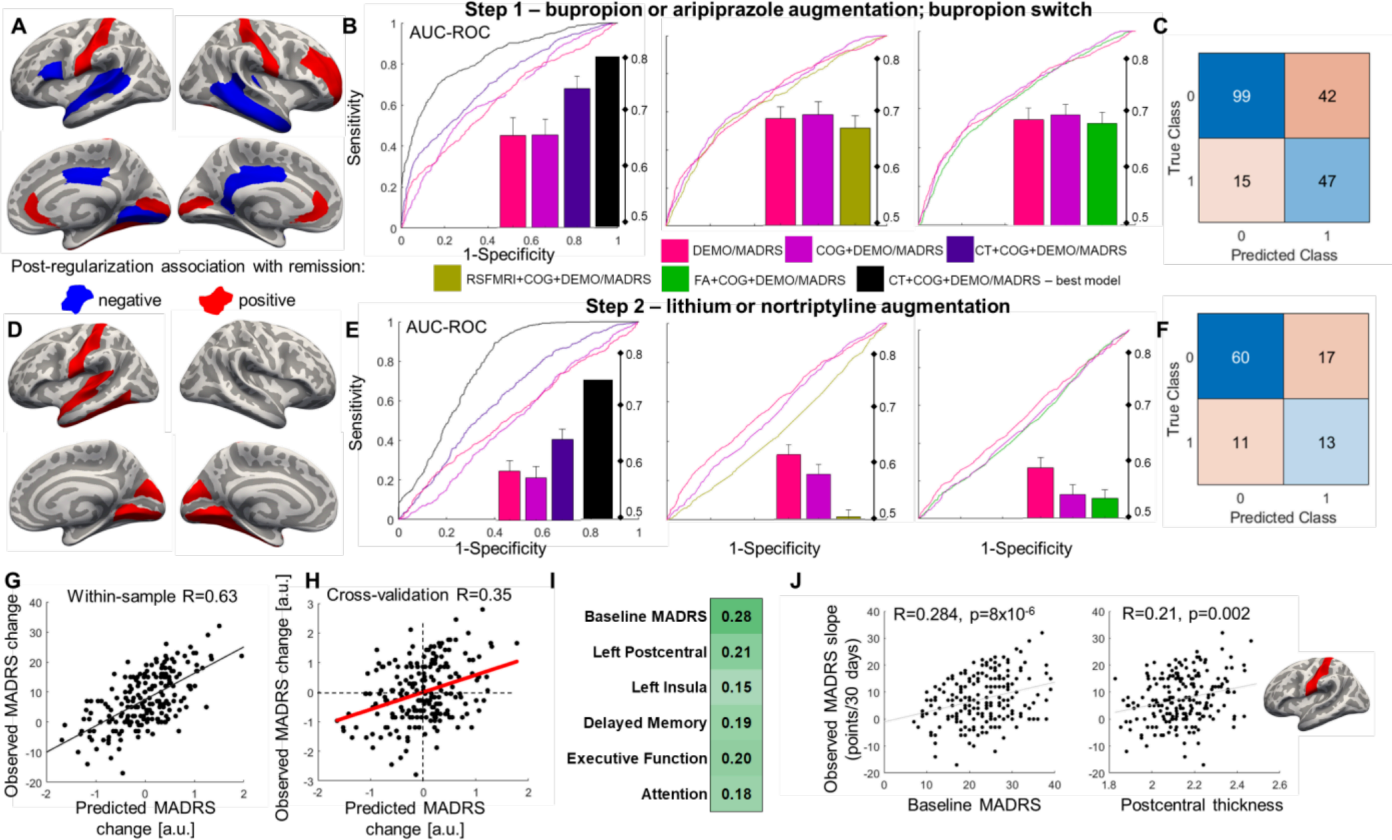
Treatment outcome prediction using both cognitive and neurobiological markers. Elastic net regression models predicting response to treatment in step 1 of the OPTIMUM trial performed best when brain structure and cognitive data were included as predictors (A, B). Cortical thickness loadings contributing to the most parsimonious model are shown in (A), whereby regions with positive loadings are highlighted in red and regions with negative loadings are highlighted in blue. Elastic net regressions predicting response to treatment in step 2 performed worse than the step 1 models; cognitive data did not improve performance in held-out data (D, E). Confusion matrices with true and false positives and negatives for the best performing models predicting remission in steps 1 and 2 are shown in (C) and (F), respectively. A partial least squares (PLS) regression including demographic, clinical, cognitive and brain structure predictors explained a significant amount of variance (39.7%) in slopes of change in MADRS scores (G), with moderate predictive accuracy in hold-out data (H). Some of the significant predictors (|Z|>3) and their correlation with the MADRS slopes are shown in (I), with a complete list included in Supplementary Table 2. Example correlations between change in MADRS scores and baseline MADRS or postcentral thickness are show in (J). MADRS: Montgomery-Asberg Depression Rating Scale.

Cross-validated elastic net regression models (Supplementary Information) showed varied prediction performance for remission (MADRS ≤ 10) in step 1 depending on the included predictors. First, we found a cross-validation AUC of 0.64 when including cognitive data alongside baseline MADRS and demographics. In the sample with both cognitive and neuroimaging data the AUC for the same model was 0.66. This increased to an AUC of 0.74 when also including 74 brain structure variables (Fig. 5A, 5B, 5C). When testing the best performing model including 16 cortical thickness variables, executive function and attention scores, and baseline MADRS as predictors, the out-of-sample AUC increased to 0.83 (specificity = 0.70, sensitivity = 0.78). Neither resting-state fMRI nor diffusion derivatives improved classification performance of remission in step 1 or step 2. In step 2, cortical thickness but not cognitive data was predictive of remission (Fig. 5D, 5E, 5F). When testing the best performing model including three cortical thickness variables and baseline MADRS as predictors, the out-of-sample AUC increased to 0.78. When operationalizing treatment response as change in MADRS scores, a PLS regression with demographic, clinical, cognitive and brain structure data achieved considerable in-sample and hold-out accuracy (Fig. 5G, 5H, 5I, 5J; PLS permutation P < 0.001).

### Sensitivity Analyses

We ran three additional FC PLS models, testing for the brain cognition associations in run 1 and run 2 separately, and in 196 individuals with mean FD < 0.5. The results of the main analysis were consistent with the results of the sensitivity analyses (Supplementary Section 5). Consistent with the whole brain analyses of pairwise connectivity of network components, seed-based connectivity analyses of the precuneus, overlapping with the posterior-medial default mode IC1, also showed that reduced connectivity with inferior parietal and inferior frontal regions and increased connectivity with entorhinal and perientorhinal cortex was associated with better cognitive function (SI Section 4). Second, we repeated the hold-out analyses by splitting the sample according to the scanner used (GE vs Siemens Prisma). Prediction accuracy was substantially lower in held-out data of a different scanner type (SI Section 6). Third, given that the cognitive and neuroimaging visits occurred after the completion of OPTIMUM treatment for some participants (SI Section 3), it is possible that treatment has also impacted these markers. Therefore, we repeated some of the treatment outcome prediction analyses, while only including participants who completed cognitive and neuroimaging visits before the end of their treatment. The prediction results remained highly consistent, with relatively strong performance of the best model in this subsample, too (SI Section 7).

## DISCUSSION

In this study, we identify distinct neural correlates of poorer cognitive function in older adults with treatment-resistant LLD. As hypothesized, we found that de-segregation between frontoparietal and default mode circuits and loss of gray matter predicted worse cognitive function. Further, worse baseline cognitive function and brain structure predicted ongoing treatment resistance, with robust cross-validation performance improvements (AUC > 0.74) obtained by adding cortical thickness predictor features.

We found that higher connectivity between components of the frontoparietal and default mode networks was associated with worse memory recall, processing speed, and executive function. Higher between-network and lower within-network connectivity, i.e. de-segregation of these brain systems, is typically found in older compared to younger adults^24–26^, is linked to worse working memory^25^ and episodic memory^24,27^ and is also found in major depression^28^ and MCI^29^. Resting-state connectivity of the posterior default mode regions and frontoparietal regions including the dorsolateral prefrontal cortex is a biomarker for better memory performance in older adults^14,15^ and posterior parietal activity is related to better memory performance^30^. Recent brain stimulation studies showed that transcranial magnetic stimulation of the precuneus showed preserved cognition^31^. Our findings support further investigation of the posterior default mode circuits as a key correlate of cognitive function and a potential intervention target for slowing cognitive impairment in LLD.

We also found that lower white matter integrity of several tracts (e.g superior longitudinal fasciculus) was specifically associated with worse Trailmaking performance. This finding is consistent with previous studies showing that reduced integrity of tracts connecting prefrontal and parietal areas, including the superior longitudinal fasciculus, are associated with worse executive function in healthy individuals^32^, in those with varying levels of cognitive impairment^33^, but also in children^34^ and young adults^35^. Similarly, faster processing speed is associated with higher FA in tracts including corpus callosum^36,37^ and average cerebral FA^38^, and cortico-striatal tracts^39^. Interestingly, FA was not significantly associated with episodic memory performance in our analysis, consistent with previous studies of white matter^39,40^. We also show that white matter hyperintensities, an index of cerebrovascular disease burden, were predictive of both cognitive function and FA scores, consistent with the theory that cerebrovascular pathology affecting white matter may be driving cognitive impairment^7^. Our findings further support the hypothesis that initial impairment in white matter in treatment-resistant LLD may predominantly impact processing speed and executive domains rather than memory and language domains.

Our follow-up analyses also identified several protective factors for cognitive function in LLD. First, education predicted better cognitive performance and higher white matter integrity. While a consistent positive effect of education on cognitive ability in late-life has solidified its role as a proxy of cognitive reserve, there is conflicting evidence for education effects on rates of cognitive decline^41–46^ and Alzheimer’s Disease^47^. In addition, brain structure centile scores also showed protective effects on cognition, consistent with previous studies showing lower brain centiles in MCI and dementia^48^ and executive function in healthy older adults^49^. Our findings are novel in that they are the first in patients with treatment-resistant LLD to demonstrate these relationships, and largely align with the literature in other late-life populations. They also provide important insights into key factors protecting against cognitive impairment for this at-risk population.

In addition to identifying brain-cognition relationships, our predictive analyses advance the search for biomarkers predicting remission in LLD in several ways. Cognitive performance and gray matter integrity predicted remission after 10 weeks of bupropion or aripiprazole treatment in the OPTIMUM trial. Patients with better cognitive function and greater rostral ACC and postcentral gyrus thickness were more likely to achieve remission. The use of neuropsychological data (along with demographic and clinical predictors) provided a reasonable AUC ranging from 0.64–0.66 (in both samples). However, the addition of neuroimaging in the model boosted the AUC to 0.74. These results showcase the utility of biomarkers in helping target specific medications to patients who are more likely to benefit from them^50–52^. Recent work by our group^53^ and others^54,55^ showed that biomarkers are less generalizable when tested out-of-trial, especially when the patient populations are different in age, severity, or other clinical features. Adult MDD biomarker studies have shown functional connectivity^53–56^ and task-based activation^52,57^ to be important predictors of remission. In older adults with considerable variability in gray matter integrity and cognitive performance, we found gray matter structure but not brain function to predict remission, potentially suggesting distinct biomarkers in TRLDD and adult MDD. Cross-trial generalizability studies^53,54^ are needed to test whether unique biomarkers apply in different MDD patient populations. Overall, our results show that neuroimaging features are a valuable biomarker addition, though early response to treatment after 1–2 weeks has been shown to be a key predictor and should be tested in future studies^53,55^.

Our study has several strengths and limitations. First, we leveraged a unique, deeply-phenotyped sample of patients with treatment-resistant LLD with advanced neuroimaging data to test for multivariate brain-cognition associations. We use a large-scale parcellation derived using a data-driven group independent component analysis from a large sample of older adults in the UK Biobank^22,58^, in line with recent evidence showing that brain activity can be parsimoniously explained by geometrically constrained brain-wide modes of brain geometry^59^. We corroborate the findings from this whole-brain approach using a seed-based FC analysis. The effect size of brain-cognition relationships identified here was moderate, although relatively strong out-of-sample generalization of the predicted cognitive scores in the FC-PLS is encouraging. Although cognitive function and gray matter structure assessments were taken at varying time intervals relative to the OPTIMUM treatment, unlike functional connectivity, these markers are more stable over time. Nevertheless, it is possible that OPTIMUM treatment has also impacted these markers. Our supplementary results support the former hypothesis, given that excluding participants with post-treatment scans and cognitive testing did not substantially change the results. Previous brain-cognition association studies in remitted LLD and MCI have found similar association levels between brain structure and cognitive function^33^. Future analyses of longitudinal data from the OPTIMUM-NEURO study will help identify neural correlates and clinical determinants of cognitive decline while also considering patients’ remission status from the clinical trial. Finally, measures of cognitive reserve are imperfect; for instance, education is strongly affected by socioeconomic status, thus presenting a limitation in the mostly white population studied in this trial. Future studies including more participants from minority groups and may uncover more cross-cultural protective factors.

In conclusion, varying levels of cognitive performance in older adults with treatment-resistant LLD have distinct neural correlates, with protective effects of education and structural brain maintenance. Our analyses lay the foundation for ongoing prospective longitudinal analyses to determine who among patients with TRLDD is at highest risk of neural and cognitive decline, and how that risk relates to treatment response vs. resistance.

## METHODS

### Participants.

All participants were enrolled in the parent “Optimizing outcomes in older adults with treatment-resistant depression” (OPTIMUM) trial, with detailed protocol, and primary outcome results recently published^1,60^, and concurrently enrolled in the present OPTIMUM-NEURO study which added MRI scans and detailed neuropsychological testing. The trial was conducted in accordance with the Good Clinical Practice guidelines of the International Council for Harmonisation and was governed by an independent data and safety monitoring board. Ethical approval was obtained from the institutional review boards of each of the five sites - Washington University in St. Louis; Columbia University; the University of California, Los Angeles; the University of Pittsburgh; and the University of Toronto. This report does not include participants from the Columbia University site due to an ongoing pause for all human subject research in the Department of Psychiatry at that site; this pause currently precludes the analyses of data for any ongoing human subject research study. All sample sizes reported exclude participants from Columbia University. Inclusion/exclusion criteria for the clinical trial are summarized in the Supplemental section and are previously published. Informed consent was obtained from all the patients before enrollment. To take part in the OPTIMUM trial, patients had to be over 60 years old and have a diagnosis of current major depression according to DSM-5 criteria which persisted despite two or more trials of antidepressants of adequate dose and duration as classified by the Antidepressant Treatment History Form (ATHF) within the current episode^61^. Patients with dementia (Short Blessed Test ≥ 10) were excluded. Treatment resistance was determined by research staff using a PHQ-9 score of 6 or higher, which was later amended to 10 or higher. In addition, patients were required to be taking one adequately dosed antidepressant. Exclusion criteria included severe neuropsychiatric conditions such as Parkinson’s Disease or schizophrenia, uncorrected sensory impairment, imminent risk for suicide, and moderate-to-severe substance or alcohol use disorder. Patients were recruited via referrals from primary care providers and psychiatrists, outreach from the trial team, automated alerts in electronic medical records and print, radio, social media, and office advertisements. Participants who had no contra-indications for MRI scanning were offered participation in the OPTIMUM-NEURO study, that was funded to evaluate the trajectories of cognitive function (focusing on memory and executive function) and brain structural and functional decline (focusing on cortico-limbic and fronto-executive circuits). The parent trial included two steps, each 10 weeks’ duration. In step 1, patients were randomly assigned 1:1:1 to a switch to bupropion or augmentation of their current antidepressant with bupropion or aripiprazole. In Step 2, patients who were ineligible for step 1, or who did not remit or otherwise benefit from their step 1 treatment, were randomly assigned 1:1 to a switch to nortriptyline or lithium augmentation.

### Clinical data.

We used the Montgomery-Asberg Depression Rating Scale (MADRS) to assess depression severity closest to the time of the initial MR scan and neuropsychological testing visit^62^, and the change in depressive symptoms due to the treatment offered through the OPTIMUM clinical trial. Finally, we used the Cumulative Illness Rating Scale – Geriatrics (CIRS-G) to quantify general disease burden^63^.

### Cognitive data.

Twelve tests of memory and executive function, verbal fluency and processing speed were included because these cognitive processes are most affected by depression, normal aging and dementia. Participants’ scores were normalized against benchmark data provided by Delis-Kaplan Executive Function System (DKEFS) and Repeatable Battery for the Assessment of Neuropsychological Status (RBANS, Supplementary Information). In the full sample with neuropsychological data (n = 397), only 3–5% of scores on each test were missing and were imputed using the mean to ensure that we could run multivariate analyses on the full sample. Cognitive status (intact, MCI and probable dementia diagnoses) was determined via adjudication following DSM-5 and NIA-AA 2011 criteria with a team of research staff, neuropsychologists, and psychiatrists based on the cognitive assessment scores and clinical presentation.

### MRI data acquisition.

We acquired high quality T1-weighted, diffusion MRI (dMRI) and resting-state fMRI sequences on 3T whole-body scanners using harmonized Adolescent Brain and Cognitive Development study protocols^64^ across the five sites (Supplementary Information).

### T1 structural data.

We used FreeSurfer (Version 6.0.0) to derive total intracranial volume, gray, white, and CSF volumes and brain structure centiles quantifying deviations from normative data in over 100,000 individuals^48^. These centiles were used as a proxy for brain maintenance^65^. In addition, we utilize FreeSurfer-derived total volume of white matter hyperintensities, corrected for total intracranial volume, and transformed using the square root function to ensure normality of the skewed distribution as an alternative index of cerebrovascular health^66^. In region-wise analyses of brain structure, we included FreeSurfer-derived cortical thickness^67^ and lateralized volumes of the hippocampus, amygdala and striatum corrected for total intracranial volume. Images with a total number of surface holes > 380 were excluded ^68^.

### Resting-state functional MRI (fMRI) connectivity data.

Two runs of fMRI data were pre-processed using fmriprep^69^; the resulting minimally preprocessed images (in the NLin6 MNI space) were denoised by regressing out 24 noise components (Supplementary Information) and smoothed with a Gaussian kernel with full-width half measure of 3mm. The first three volumes were discarded to reach steady-state equilibrium. Demeaned and normalized timeseries from the two timeseries were concatenated, with partial least square (PLS) results from individual runs available in the Supplementary Information. Participants with mean framewise displacement FD < 0.7 were kept, and sensitivity analyses with a more stringent threshold of mean FD < 0.5 are presented in the Supplementary Information.

### Diffusion weighted imaging data.

Diffusion image pre-processing followed previous studies^70^ and included (i) brain masking (using AFNI and MRtrix3 dwi2mask), (ii) motion and eddy current correction (FSL eddy), and (iii) susceptibility distortion correction (BrainSuite BDP). We used 3D slicer to fit DTI tensors and reconstruct white matter tracts via deterministic unscented Kalman filter tractography^71^ (https://github.com/SlicerDMRI). Next, we ascertained individual white matter tracts by clustering fibers and applying supervised groupwise registration to the ORG (O’Donnell Research Group) atlas^72–74^. Finally, we analyze the fractional anisotropy (FA) as a measure of tract integrity. Among 73 reconstructed tracts, we excluded tracts with more than 3% unusable data and imputed missing data in the remaining 62 tracts.

### MRI data harmonization and quality control.

Batch and site artifacts can present a challenge in multi-site trials such as OPTIMUM-NEURO. The most important mitigation step is prospective harmonization, which was done here via the use of ABCD protocols at all five sites. In addition (Supplementary information), we batch normalized the data for age, sex, and site for both functional connectivity and diffusion data using ComBat^75^; we also included average head motion in the harmonization of the fMRI data. Visual quality control of each data modality output was completed, and participant scans were excluded when anatomical segmentation of gray or white was inadequate, too much motion was present or registration between modalities was inadequate.

### Statistical analyses.

We used three partial least squares regressions to identify latent variables capturing multivariate relationships between functional connectivity and cognitive function (FC-PLS), between fractional anisotropy and cognitive function (WM-PLS) and between gray matter structure and cognition (GM-PLS). Model significance was tested using permutation testing following previous studies^14,76^ (n = 5,000).

We z-scored the predictor matrix X and the outcome matrix Y (X = 211 × 210 and Y = 211 × 12 in the FC-PLS; X = 219 × 62 and Y = 219 × 12 in the WM-PLS; X = 212 × 74 and Y = 212 × 12 in the GM-PLS). PLS returns a set of latent variables that attempt to maximize the covariance between the PLS scores summarizing X and Y. PLS scores are a linear combination of the predictor variables (X) and component loadings. We used bootstrapping (n = 5,000) to identify predictors that showed robust contributions to the each PLS latent variable. A threshold of |Z| > 3 was chosen to identify the most robust connectivities significantly associated with cognitive performance (see above). We correlated the latent brain scores (XS) with the cognitive tests and applied Bonferroni correction to identify cognitive tests significantly associated with each latent variable.

### Robustness analysis in held-out data.

To evaluate the robustness of PLS performance in each of the sites, we split our participants into four subsamples, one for each included site. We used three of these subsamples as training data and the remaining subsample as test data. We applied the PLS beta regression coefficients obtained in the training sample to the test sample and correlated the observed cognitive data with the predicted cognitive data to assess PLS performance in predicting cognitive function in held-out data. Instead of keeping all cognitive tests, we created a composite cognitive variable using a principal component analysis of variables significantly associated with FC, tract integrity, and brain structure in the FC-PLS, WM-PLS, and GM-PLS respectively. All code is publicly available at https://github.com/peterzhukovsky/brain_cognition_TRD.

### Prediction of treatment outcomes.

We used regularized, cross-validated elastic net logistic regressions to predict remission (MADRS ≤ 10) to approximately 10 weeks of acute antidepressant treatment in the parent OPTIMUM trial. Step 1 and step 2 were considered as separate studies. On each of 100 iterations, we split the data into training and test datasets, featuring 20 randomly selected participants in the test dataset for step 1 and featuring 20 randomly selected participants in the test dataset for step 2. This approximately corresponds to 8-fold and 3.5-fold cross-validation in the outer fold. On each iteration, we used 10-fold cross-validation to train the elastic net models in the inner fold. Area-under-the-curve (AUC) measures were then used to assess model performance in the held-out test data. Three sets of prediction models were run, each including demographic, clinical, cognitive data and one of the three imaging modalities. These models were run in the larger sample (n = 397) including cognitive data, clinical data, and demographic data. Then the model was re-run in the sample (n = 234) that also included neuroimaging to determine the potential improvement in the AUC when adding neuroimaging. Following this iterative process, we selected the most parsimonious model that included predictors surviving regularization in over 95% of models ran. We then tested the performance of this most parsimonious model in an even 8-fold and 5-fold split of the step 1 and step 2 data, respectively, presenting confusion matrices of this model showcasing the true positives and negatives as well as false positives and negatives. In addition, we have used a PLS model predicting MADRS change (absolute difference in MADRS score between baseline and the end of last step completed) from clinical, demographic, cognitive and brain structure data. More information on predictive modeling can be found in the Supplementary Information.

## Figures and Tables

**Figure 1:**
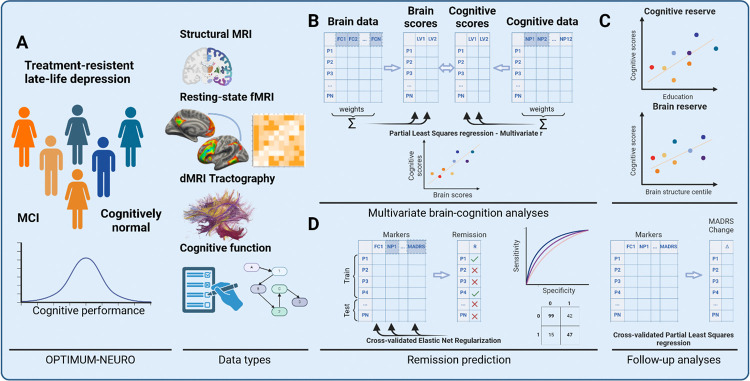
Overview of study design and analyses. We included participants with various levels of cognitive function, MRI and clinical data in our analyses (A). Across all participants, we conducted three multivariate partial least squares (PLS) regressions (B). The first PLS model tested for associations between functional connectivity (FC) and cognitive function; the second PLS model tested for associations between fractional anisotropy, a measure of white matter tract integrity, and cognitive function; the third PLS tested for associations between gray matter and cognitive function. A total of 6 cognitive domains were included. We followed up the PLS analyses by testing for associations between PLS brain and cognitive scores with education and brain structural reserve (C). In addition, in a separate set of analyses we used cross-validated logistic regression models to predict patients’ remission status using clinical, demographic, cognitive and neuroimaging markers (D). Area-under-the-curve and confusion matrices were used to assess and visualize model performance. The time at which ‘baseline’ neuropsychological and MRI assessments were completed relative to the parent OPTIMUM trial is shown in the bottom panel, with more details available in Supplementary Section S2. Abbreviations: OPTIMUM-NEURO: Optimizing Outcomes of Treatment-Resistant Depression in Older Adults; MCI: Mild Cognitive Impairment; Montgomery-Asberg Depression Rating Scale; LV: latent variable; P1-PN: participants 1:N; NP: neuropsychology and MRI assessment

**Table 1:** Overview of sample demographics.

OPTIMUM-NEURO Sample Characterization				
Demographic & Clinical Variables of Interest	Characteristics of Sample with Neuropsychological Data		Characteristics of Sample with both Neuropsychological and MRI data	
N	397		234	
Age (Years)	68.2	(5.9)	67.7	(5.4)
Female Sex (N)	268	68%	167	71%
Race (W/AA/A/NA)	354/28/8/7		209/16/4/5	
Ethnicity (H/Non-H)	375/22		228/6	
Education (Years)	14.8	(2.5)	14.7	(2.7)
MADRS score	19.4	(8.9)	19.7	(9.1)
Diagnosis (NC/MCI/DEM)	178/197/13		117/102/6	
ATHF score	8.0	(2.8)	8.2	(2.8)
CIRS-G score	8.3	(4.5)	8.9	(4.2)
MoCA score	24.8	(4.7)	24.9	(4.7)

Means and standard deviations are shown. The following diagnoses were made by adjudication among neuropsychologists: NC: Normal Cognition; MCI: Mild Cognitive Impairment; DEM: probable dementia. W: White Caucasian, AA: African American, A: Asian, NA: not answered, H: Hispanic, Non-H: non-Hispanic; MADRS: Montgomery-Asberg Depression Rating Scale; ATHF: Antidepressant Treatment History Form; CIRS-G: Cumulative Illness Rating Scale - Geriatrics; MoCA: Montreal Cognitive Assessment. CIRS-G provides a measure of total medical burden, where a score of 8 indicates roughly 4 moderate-level conditions (eg hypertension). CIRS-G provides a measure of total medical burden, where a score of 8 indicates roughly 4 moderate-level conditions (eg hypertension). An ATHF score of 8 or more indicates that participants failed at least two adequate antidepressant trials. There were no differences between the N = 397 who completed neuropsychological testing in the context of the clinical trial, with the N = 234 who successfully completed both neuropsychological testing and MRI.

## Key points

### Question

Older adults with treatment-resistant late-life depression (LLD) are at significant risk for cognitive impairment. The relationship between treatment response and cognitive function in this population is not well-established. We examined neural correlates of executive and memory function, and their relationship with prospective treatment outcomes.

### Findings

In older adults with treatment-resistant LLD, higher connectivity between components of the default mode network and the frontoparietal network and within the frontoparietal network predicted worse cognitive function across memory, executive function and language, while white matter circuit impairment predicted worse executive function. Lower cortical thickness and hippocampal volumes were also associated with worse memory and verbal fluency. Higher education duration was associated with better cognition. Both poorer cognitive performance and loss of gray matter predicted lower likelihood of remission to 10 weeks of protocolized antidepressant treatment.

### Meaning

Our findings suggest that domains of cognitive function in treatment-resistant late-life depression have distinct neural correlates related to white matter and functional connectivity respectively, with education operating as a protective factor. We also identify potential neural and cognitive markers of ongoing treatment resistance.
